# How Can Service-Learning Shape the Political Perspectives of Pre-Service Teachers? A Program in the Field of Physical Education

**DOI:** 10.3390/ijerph19159175

**Published:** 2022-07-27

**Authors:** María Maravé-Vivas, Celina Salvador-Garcia, Jesús Gil-Gómez, Teresa Valverde-Esteve, Ricardo Martín-Moya

**Affiliations:** 1 University Jaume I of Castellón, 12006 Castelló de la Plana, Spain; marave@uji.es (M.M.-V.); salvadoc@uji.es (C.S.-G.); jegil@uji.es (J.G.-G.); 2 Department of Didactics of Music, Visual and Body Expression, University of Valencia, 46022 València, Spain; 3 Department of Physical Education and Sport, Faculty of Education and Sports Sciences, Campus of Melilla, University of Granada, 18011 Granada, Spain; rmartinm@ugr.es

**Keywords:** university service-learning, teacher education, sport, civic engagement, social justice

## Abstract

Active and democratic citizenship promotion has become a critical challenge for higher education, and civic engagement is a fundamental axis not only in education but also in fostering democratic systems. Consequently, teacher educators held a prominent role through their own teaching practices to generate contexts promoting critical thinking, skills and attitudes. The aim of this study was to analyze the learning related to the political dimension developed by pre-service teachers (n = 123) after participating in a Service-Learning program through physical education with children at risk or/and student with educational needs. This research followed a qualitative research approach and we based the analysis of reflective diaries on Gorham’s (2005) categories regarding political learning: Critical political thinking, Interest in politics, Deliberation and Political judgment. The findings show a development in learning such as civic attitudes, critical political thinking, awareness of social justice problems, increased civic compromise and responsibility. The Service-Learning program, therefore, may have been an adequate option to develop pre-service teachers’ learning related to a political perspective. Therefore, these findings let us understand how Service-Learning may foster equity and social justice among future teachers.

## 1. Introduction

Active and democratic citizenship promotion has become one of the most critical challenges for European Higher Education Institutions [1]. Without any doubt, education and civic engagement are fundamental axes in democratic systems. Within the physical education (PE) context, this is particularly pertinent, since we live in an era with growing concerns about the impact of neoliberal globalization and the precariousness of society. Therefore, efforts should be made to promote equity, democracy and social justice in this field [2]. In this sense, universities held a pivotal position to foster these elements. In fact, a decade ago, there was an emphasized surge focused on civic engagement promotion in higher education [3]. At that point, higher education institutions set off on a journey aimed at renewing their civic mission, showing many scenarios in which students can develop the Sustainable Developmental Goals (SDG) proposed by the European Union in the field of PE [4,5]. Thus, universities eagerly embraced adjustments and changes within their strategic plans to respond to unprecedented demands such as educating informed and responsible citizens as well as promoting a civic mindset among their students [6,7].

The teaching processes at the university level challenge students and professors to dominate skills far beyond written or oral expression. For instance, ‘critical thinking’ emerges as a salient capacity to be promoted among undergraduates [8]. In the political context, ‘critical thinking’ allows citizens to think and judge governmental programs, thereby triggering students to become closer to the political language [9]. Furthermore, Astin et al. [10], add the commitment to activism or radical understanding, while Assendelft [11] refers to the interest in politics or the empowerment for being responsible citizens. Concurrently, specific literature has pointed out that, during the last years, millenials have apparently ignored politics, especially in comparison to previous generations [8,9,11].

Universities may act as levers for change in order to promote ‘critical thinking’ related to political awareness among their students. There are a number of possibilities to tackle this enterprise, and one of them is Service-Learning (SL). This is “a course-based, credit-bearing educational experience in which students (a) participate in an organized service activity that meets identified community needs and (b) reflect on the service activity in such a way as to gain further understanding of the course content, a broader appreciation of the discipline, and an enhanced sense of civic responsibility” [12] (p. 112). SL can contribute to the inclusive education from the field of PE, in the line of the SDG [4,5]. Among many other aspects, according to literature, SL may increase students’ interest in politics [13]. This stems from the fact that, when participating in SL, students become ‘expert citizens’ directly engaged with public affairs [9]. In fact, according to Gorham [9], SL may lead participant students to acquire ‘political thinking’ including dimensions such as increased interest in politics, reflecting on their public and private interests, prudent participation and deliberation, listening politically, and judging the political world as intelligent performers.

SL participation promotes the alliance among members of the community, and reinforces the actions carried out with reflective readings and discussions in order for this methodology to be successful [11]. Furthermore, SL promotes transformative learning [14] by building community and making significant changes to improve this scenario [15]. Since meaningful learning is promoted through the linkage of observation of real social problems, reflection upon them and derived action [16], SL contributes to political learning acquisition by increasing the students’ ability to recognize and participate actively to wrestle social injustices [14,17]. In addition, SL is rooted in ‘experiential learning’ [18], and it firmly encourages students’ reflection [19]. Therefore, undergraduates may develop their critical thinking by reflecting on their experiences in relation to political perspectives. This may lead towards an increased sensitivity towards the understanding of the political attitudes, as well as an increased awareness of their critical role as active social agents, which, at the same time, leads to an enhanced attitude towards activism or response to first-hand problems [20].

According to Butin [21], SL promotes students’ learning from a number of perspectives. Particularly, this author established four different learning dimensions on which SL may have an impact, one of such being precisely the “political perspective”. This political dimension is focused on students’ understanding of society and also considers the development of attitudes and behaviors encouraging social participation from a transformative lens. Delving into political learning, Gorham [9] considers that SL fosters political thinking and, in fact, he asserts that this pedagogical method may be connected to four epistemological objectives in this respect, namely, critical political thinking, interest, deliberation, and judgment.

All teaching is shaped by the surrounding political context. Thus, it is often considered that teaching is a political act [22,23]. In this sense, critical pedagogy emerges as a crucial approach, since it conflates equity, justice, and social principles as well as values in order to promote reflection upon acts of domination and the beliefs and practices that generate them. In the PE field, Kirk [2] considers that critical pedagogy is ‘concerned with the organization and alignment of curriculum, teaching, learning and assessment in ways that render physical education inclusive, fair and equitable as an embodied experience’ (p. 101). Therefore, critical pedagogy is aimed at developing civic and political learning, which is a learning dimension highly relevant for pre-service teachers, who are the participants of this study.

Pace and Kasumagić–Kafedžić [24] assert that teacher education is a key stage to promote pre-service teachers’ skills. They particularly highlight that this stage may strongly influence their capacity for educating civic and democratic citizens, since it may provide pre-service teachers with pedagogical experiences and tools that enable them to embed democratic and transformative approaches to their future teaching practices. Consequently, teacher educators held a prominent role through their own teaching practices, as they are expected to generate contexts promoting critical reflection and awareness. Harry and Salvador [25] affirm that teaching is an inherently political act. Therefore, they highlight that teacher educators should be conscious of their role in the public discourse and how they tackle the political dimensions of teaching with pre-service teachers. Moreover, McCardle et al. [26] consider that teacher education should take a more powerful role in the socialization of pre-service teachers, leading them toward educational activism. Against this backdrop, the project examined in the present study was based on SL, since this methodology could be instrumental to build political knowledge and thinking among pre-service teachers from a critical pedagogy approach [27].

To our knowledge, specific literature on SL in teacher education analyzing pre-service teachers’ political learning and thinking is scarce. For example, Iverson and James [28] indicate that students develop learning related to political dimensions, such as their civic-political identity, the understanding of citizenship and responsibility for their own actions. Similarly, according to Cho and Gulley [29], SL may enhance students’ intercultural competence and civic responsibility, while Tan and Soo [30] assert that pre-service teachers’ self-concept, collaborative and communicative skills are also increased. In the specific field of PE, some studies conclude that SL comes with learnings related to the development of civic attitudes and skills [31], civic engagement [32,33] or a greater awareness of social justice related problems [34]. Whereas, a previous study in the line of the one we present, used a qualitative approach to analyze the critical and inclusive approach of the Preservice PE teachers after their participation in a SL program with children with Special Education Needs (SEN) [19]. In addition, a qualitative study focused on PE pre-service teachers examined the technical, cultural, political and poststructural perspective after their involvement in experiential learning through SL [35]. Nevertheless, we did not find any study focused on examining political thinking and learning specifically. Therefore, the aim of this study was to analyze the learning related to the political dimension developed by the participant pre-service teachers after participating in a SL program in the context of the PE.

## 2. Materials and Methods

### 2.1. Research Design

This investigation followed a qualitative research approach design [36], aimed at understanding the complexity of the social phenomena based on the participants’ perspectives [37]. This approach allowed us to carry out a holistic and in-depth analysis of the phenomenon we aspired to examine (SL program) within its specific context [38]. The research question to be answered in this study was: What political-related learnings are developed by pre-service teachers after participating in a SL program?

### 2.2. Participants

The participants of this research were 123 pre-service teachers enrolled in the subject “Fundamentals of Body Expression; Motor Games in Early Childhood Education”. This is a compulsory course in the second year of the Degree in Early Childhood Education of the Jaume I of Castellón University.

### 2.3. SL Program

A general SL program was implemented in the aforementioned subject. Within this general program, a number of projects were applied using a direct service modality. This type of SL entails a direct relation between students and service receivers in a real context [39]. In this case, all projects had to revolve around the contents related to body expression, motor skills and games, which are included in the curriculum of the second cycle of the Early Childhood Education stage in Spain. Table 1 shows an overview of the SL program, since it presents the contents of the subject, its academic objectives as well as the service aims.

**Figure 1:**
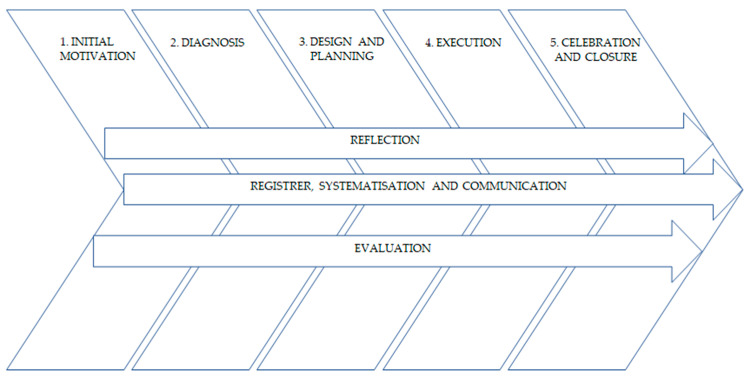
Phases of a Service-Learning Project. Source: model proposed by CLAYSS [40].

Students were grouped to design and carry out their projects in groups of four to six students. Each group could work with a different social organization, each of them attending a specific type of population (the specifications regarding each social organization are clearly described in the subsequent section of the paper). In order to properly carry out a project, it is necessary to follow some guidelines that the groups had to apply in their specific contexts. Specifically, their projects followed the model proposed by CLAYSS [40] (Figure 1).

In the diagnosis stage, each group had to identify the motor and body expression needs of the children they were going to work with. The contents related to motor and body expression skills had been developed in class previously, both theoretically and in praxis. Based on this previous experience and knowledge, pre-service teachers had to establish the specific objectives for the sessions of their project. After this, they specified the contents to be worked in the sessions that would be carried out with the children.

Concurrently, teacher educators carried out a number of procedures to help pre-service teachers organize, develop and sequence their projects properly. On the one hand, university students had to write a reflective diary. This tool facilitates the organization of the stages of the program, since pre-service teachers had to reflect and note down all the information they were gathering along the project implementation. This reflective diary, thus, was critical foster reflection among pre-service teachers, helping them to constantly adjust the sessions in terms of structure, difficulty, sequence, resources, space, time, etc. On the other hand, teacher educators guided the students through a number of group tutorials. In these meetings, they revised pre-service teachers’ work and shared some feedback about it. Furthermore, at the end of every session, the group of students had to carry out a final and shared reflection, to which volunteers and teacher educators were also invited.

### 2.4. Children Receiving the Service and Social Organizations

The different social organizations with which the program was implemented attend to vulnerable groups with different disorders, ages and features. All the participant children though, had the communicative, socialization and motor areas affected to an extent. In addition, each of these entities has different personnel, material and economic resources, so they have specialists with whom the students could interact during the development of their projects.

In any case, regardless of the social organization in which the project was developed, the phases and objectives of each group were the same: to respond to the null offer of leisure-sports activities existing in the city for children with functional diversity, both in the public and private institutions. The different entities that were part of the SL program and the characteristics of the children they attend are specified below:

1. Association of Parents of Children with Autism Spectrum Disorder of Castellón (APNAC). This association works with children diagnosed with autism spectrum disorder (ASD). It was founded with the purpose of providing an immediate and adequate response to the needs of children with ASD and their families in the province of Castellón. It aims to collaborate with and assist families, since they consider that, by promoting the well-being of the family, the basic pillar of ASD children, they are also facilitating the children’s well-being.
2. Penyeta Roja Special Education Center (PENYETA). It looks after children with functional diversity who cannot be enrolled in ordinary schools. They have severe and permanent special educational needs, derived from functional diversity or serious behavioral disorders. The main objective of this organization is to ensure that all children reach their maximum development, regarding the social, intellectual, cultural and emotional capacities.
3. Tombatossals Early Childhood and Primary School of Castellón de la Plana (TOMB). This school has a specific communication and language unit, which is a special education classroom located in an ordinary school aimed at giving an adequate educational response to students with ASD. Thus, it allows for individualized attention and promotes inclusion of ASD students in ordinary classrooms.
4. Asperger’s Association of Castellón de la Plana (ASPERGER). It deals with children diagnosed with ASD. It is an Association for families and people related to Asperger’s Syndrome from the Province of Castellón. This organization defends the rights and needs of people with Asperger Syndrome as well as their families. Thus, it develops and creates resources aimed at ensuring a better quality of life for children with ASD and their families. Its mission is to promote the effective inclusion of people with Asperger Syndrome in all areas so that they can enjoy life, developing their possibilities and abilities to the maximum extent possible.
5. Association of Parents of People Affected by Attention Deficit and Hyperactivity of Castellón (APADAHCAS). This association aims at seeking intervention mechanisms that help improve the adaptation difficulties of children with Attention Deficit and Hyperactivity. This social organization tries to guide, advise, investigate and collaborate in educational and scientific settings in relation to Attention Deficit and Hyperactivity disorders.
6. Cervantes Early Childhood and Primary Education School of Castellón de la Plana (CERVANTES). It is a public school that does not specifically attend students with functional diversity, like some of the previous ones, nor does it have a specific unit. However, this school is characterized by great cultural diversity. Therefore, a wide spectrum of religions, origins, cultures and beliefs are gathered in this center. According to the teachers working there, this fact brings out many problems among the students. This is coupled with the fact that these students do not have the necessary resources to develop the motor skills through extracurricular activities and the school has deficient facilities and resources to develop PE.

### 2.5. Instruments

Reflective diaries were the main instrument used to collect data. Pre-service teachers had to complete their diaries throughout the implementation of their project, before and after each session, and when the program finished. These reflective diaries were based on the “CApSa’’ SL program, of the University of Valencia. They had two different parts: one to be completed in group and another individually. First, the group of students had to describe the sessions they had designed and implemented, specifying the modifications and adjustments that were carried out. The difficulties that might have emerged as a group (i.e., development of the sessions, group coordination) were also to be described. Subsequently, each student had to reflect individually upon the following issues:

- Personal difficulties experienced during the different stages of the project.
- Emotions and feelings experienced, their personal involvement, achievements and expectations.
- Evolution of these feelings and emotions along the project.
- Experience and learning based on the four perspectives of Butin [21].
- Most relevant conclusions they could draw once the project had finished.

Reflective diaries were anonymized and coded. Each diary was given a number, which is accompanied by the acronym of the entity with which the group had been working. These codes appear within parentheses after the quotes used in the results and discussion section. For example, (ASPERGER_3).

### 2.6. Data Analysis

Bearing in mind the objective of the present study, we adopted an interpretative approach to data analysis. Specifically, following accepted recommendations for qualitative analysis of narrative data, a double procedure was applied, from inductive to deductive and back again [41]. Thematic areas used to perform the qualitative analysis were defined by Gorham’s [9] categories on political learning (Critical Political Thinking, Interest, Deliberation, Judgment). A multiphase analysis was carried out based on an initial open-coding phase and a second axial coding phase [41]. In the first phase, we identified the relevant information related to Gorham’s [9] categories. Therefore, the coding process was combined with the process of analysis [41]. In the second phase, we searched for additional data that could be relevant to answer the research question and could help us understand the information gathered in the previous phase. To do so, we moved between inductive and deductive reasoning, and two iterations were carried out before engaging in a member checking process, which consisted of providing the participants with the opportunity to confirm their statements and make new contributions if they so desired.

In order to establish the trustworthiness of the results, the following procedures were adopted [42]: credibility (researchers triangulation), transferability (exhaustive gathering of the data and clear description of the context of the study), dependability (description of the processes) and confirmability (data transcription, reflection upon the results and limitations identification). Furthermore, this research followed the ethical guidelines established in the Declaration of Helsinki [43] and the research team collected informed consents and all data were anonymized [36].

## 3. Results and Discussion

This section shows the findings related to Butin’s [21] political perspective. To do so, we present them following the four categories established by Gorham [9] regarding political learning gained through SL. Each category is accompanied by some extracts to evince the learnings acquired and the discussions concerning these findings.

A. *Critical Political Thinking*

The first category of the analysis is called ‘Critical Political Thinking’. In relation to this, Gorham [9] establishes that effective SL programs are expected to provide students with opportunities that encourage them to assess their experiences as citizens. Therefore, SL participants should think critically about politics, or reflect upon the efficacy and morality of government programs, among other aspects. In relation to this category, the pre-service teachers who participated in the SL program could carefully think about how the government allocates the budget. University students considered that education emerges as a key factor to improve society. However, budget allocations tend to disregard this area and, instead, they are directed towards other aspects that are less relevant from their point of view.

*In my opinion, the government allots great sums of money to unnecessary things. They should focus more on education, since it is a critical factor to enable social and citizenship improvement.*

(TOMB_31)

Pre-service teachers could visit different social organizations, where they developed their SL projects. Therefore, students could see the actual spaces and resources these entities have. In fact, several groups of pre-service teachers had to develop the PE sessions at the university because the social organization with which they were collaborating did not have adequate facilities to carry them out. Furthermore, awareness was raised regarding the relevant role of these associations not only for the children but also for their families, coinciding with what reported by Chiva–Bartoll et al. [35]. This fact makes pre-service teachers point out the social value entailed by these institutions, and they even defend that larger budgets should be allocated in this type of issue [9]. According to university students, providing these organizations with greater sums of money would help them ensure better attention to the children and their particular necessities.

*I think that there should be more associations for children with autism spectrum disorder. And these should be given more money. Their facilities are inadequate and scant, and their resources are insufficient. If the government allocated larger budgets for these associations, much more actions could be implemented to help these children.*

(APNAC_5)

Nevertheless, university students’ discourse did not only call for increasing budgets, but they also highlighted the need for greater attention towards the children. Pre-service teachers express that children have the right to engage in valuable life experiences. In addition to this, university students also show an awareness regarding the relatives and volunteers’ relevance for the associations to be operative. As a consequence, they are concerned because all the responsibility and work for these associations to continue running rests in these groups of people.

*Another aspect that I would like to bring up is that (…) these associations should be supported by the government. Instead, they are ignored and only the relatives and volunteers are trying to improve the children’s lives.*

(APNAC_22)

As it may be seen in Figure 1, which shows the phases of the SL projects, reflection emerges as a fundamental process. Critical reflection has been promoted through group tutorials and post-session feedback. These processes enabled students to develop critical thinking and understanding; in line with previous studies on SL applied in the field of PE [19,35]. Developing this type of thinking is not only beneficial for the context of the program, but also for undergraduates’ future teaching practices. In fact, according to Maloyed [8], this is a prominent capacity that must be promoted among university students.

B. *Interest*

The second category of analysis focuses on ‘Interest’. In this respect, Gorham [9] asserts that SL may foster interest among students by increasing their motivation towards paying attention to politics, helping them understand their own interests, and exposing them to the civic nature of self-interest. Thus, interest may be promoted from different perspectives, but all of them should comprise the domain of being open-minded and showing a motivation toward ideas and love of learning [44]. An example of this is shown in the following extract, in which a student expresses that:

*I have seen that, nowadays, society is careless of this topic (education of children with SEN). These children are being excluded and marginalized. This hurts me terribly, and I would like to help them to the extent possible by raising awareness, at least, among the people who are part of my closest context.*

(APNAC_19)

The SL program led students to deal with unfamiliar situations and contexts. Therefore, they could become aware that the children with whom they were working are sidelined by society. Thus, this pre-service teacher showed an interest toward this situation and highlighted her willingness to help. This student understood that her single action may be relevant by making others aware of this set of circumstances. Such a learning is related to previous literature showing that SL promotes civic attitudes and skills development [31,45]. Learning in this respect is relevant since these skills respond to the need of enhancing the scant social participation and compromise shown by young people [46].

Despite the relevance of interest skills related to political perspectives, this category was the least acknowledged by the pre-service teachers participating in this study. Interest may be promoted by choosing adequate teaching methods and pedagogical activities [47], and SL might be an appropriate choice in this respect according to the ideas shared in the aforementioned extract. Nevertheless, even the best actions do not necessarily entail an increase of interest and engagement among students [48]. In fact, sometimes, higher education students just focus on the completion of a task, but the activity itself is not experienced as engaging [49]. This is probably because the experience of interest tends to be elicited by each students’ latent disposition [50] and a number of factors might affect each pre-service teacher’s perception of interest from a political viewpoint.

In addition, it is worth noting that this SL program was not applied within a subject related to the field of politics nor was it aimed at raising political awareness and interest specifically. Therefore, we could argue that even a generic SL program, aimed at meeting the needs of at-risk populations, might promote an increase of political interest among a few students despite the fact that it was not its main objective. Similar results were observed by Chiva–Bartoll et al. [35], who showed that students detected the necessity of formation in order to include people after their involvement in a SL program in the PE subject. This type of side effect coming with SL has been previously reported, for instance, in relation to less gender-biased conceptions of PE [51].

C. *Deliberation*

Gorham [9] considers that ‘Deliberation’ is not only an epistemological skill but also a way of binding communities together. Thus, in this respect, the category focused on deliberation deals with the ability of voicing opinions and actively listening to others’ opinions regarding political issues. The findings of this study show that pre-service teachers heightened their awareness of the problems that a number of families suffer. According to the ideas shared by the students, many families do not have the necessary resources for their children to be adequately attended to in extra-curricular activities. Bearing in mind that meaningful learning occurs when students are enabled to observe real social problems [16], SL might contribute to political learning acquisition by increasing the pre-service teachers’ capacity to recognize and act to tackle social injustices [14,17].

*They (the relatives of the Special Education Needs children) cannot hire anyone to take care of their children. For this reason, we provide them with this service. In this way, these children can stay at the school and play while we develop teaching-related skills that will be basic in our future. Moreover, participating in this program lets us acknowledge social problems and try to solve them.*

(CERVANTES_1)

Similarly, previous literature on SL highlights that university students could build awareness of social justice-related problems [34,52]. However, besides of this, the extract highlights the student’s willingness to find an answer to these complexities. In fact, more students positioned themselves as social active agents.

*I have been able to reflect upon the relevance of everyone’s increasing their commitment. All the citizens have much to contribute to enhancing and enriching the community where we live. In fact, it makes no sense lifting a discourse towards solidarity. What does make a contribution is to show compromise and responsibility. As a consequence, I can say that I have a major commitment to community needs. This experience (SL) has led me toward redesigning my future plans and, currently, I am willing to keep on actively participating with different organizations or through volunteering activities.*

(PENYETA_13)

As it may be perceived, pre-service teachers understood how their participation can enhance education in their social community. In this sense, SL might be an effective tool to respond to the demands posited by authors such as McCardle et al. [26], who assert that teacher education should promote students’ socialization and educational activism. Furthermore, pre-service teachers in this study expressed that SL helped them to increase their commitment towards their social community and were eager to continue contributing to its improvement. In this sense, Adler and Goggin [53] establish that civic engagement consists of promoting active citizenship in order to help enhance the lives of other people or to help shape a better future for the community thus, being connected to the empowerment for being responsible citizens [11]. Regarding SL, Cho and Gulley [29] assert that this pedagogical method may increase students’ civic responsibility, while Richards et al. [32] and Ruiz-Montero et al. [33] suggest that it may increase civic compromise. All these points support the findings of the present study, since the participants evinced ideas in this respect.

*Another concern I felt after participating in the SL program was related to the people with any type of disability (…). Where and how will they work? Recently, they have more opportunities thanks to the government’s aid for those people who hire them. Unfortunately, I think that many of these people will not be provided with sufficient resources to truly engage in working and social life. And, in my opinion, this is really unfair.*

(PENYETA_12)

The deliberation led pre-service teachers to worry about the children with whom they had been working and to demand more budgetary help, as we had mentioned before. In addition, university students reflected on the future of these people and call into question their inclusion in basic situations of their lives. Although this interpretation stems from a profound awareness of social justice problems that we mentioned in previous sections [34,52], this extract goes deeper, since it evinces an actual change entailed by SL participation. Particularly, this student shared a renewed perception toward service receivers, in line with previous studies in which students worked with at-risk children [54,55,56].

Moreover, pre-service teachers reflected upon the relevance of proper teacher education to adequately attend to children with SEN.

*Teachers should be better trained, and they should be provided with more resources and tools to properly deal with these pupils.*

(APNAC_23)

This viewpoint is related to previous studies concluding that PE teachers do not feel sufficiently prepared to properly attend children with functional diversity in their lessons [57,58,59]. These studies point to teacher education to be key in this respect. Thanks to the direct experience provided by the SL program, pre-service teachers could gain awareness about the relevant role of teachers to foster inclusion, regardless of the capacities of the students. In this sense, Pace and Kasumagić–Kafedžić [24] assert that teacher education is a key stage to foster key competences among future teachers. In fact, according to these authors, pre-service teachers should not only possess civic and democratic competences, but also promote them among their future students.

D. *Judgment*

The fourth category of the analysis refers to ‘Judgment’. In this respect, Gorham [9] considers that SL may nurture political opinions among students, by placing them in public spaces instead of within an ordinary classroom. In this new scenario, they are expected to discuss ideas and reflect on their learning. Engaging in the SL program let pre-service teachers analyze the situation of the service receivers and reach the conclusion that not only should their needs and inclusion be addressed by the educational system, but also by other institutions and from a social and political perspective. In addition, university students could reshape their discourses thanks to their experience. Particularly, they now perceive difference and diversity from a positive viewpoint. In fact, they asserted that these differences should be made visible because it may be constructive and enriching for the society.

*I now realize that this group of children must be adequately attended by the educational system, the society and the politicians. Inclusion and diversity are not independent from the general societal system. As a consequence, not only the educational system should respond to their (children with SEN) needs, but also the rest of the society. Everyone must make visible and take into account these groups of people, as well as bear in mind their differences acknowledging the positive part of this distinction.*

(APNAC_3)

An improvement in the disposition and open-mindedness regarding diversity [60], and an attitudinal shift toward special educational needs have been previously reported in teacher education in the PE field [61]. Another pre-service teacher alluded to her own social concerns and those of the society. In fact, she considered that, from a general perspective, citizens worry about trivial issues instead of focusing on relevant social problems that must be faced and require direct action and resources.

*Nowadays, we live in a society that considers a critical problem to be something such as an insufficient number of followers in Instagram or Twitter. Therefore, real problems, such as diseases or disabled people, who need someone else’s help, are disregarded. For example, children with Special Education Needs need people to take care of them because their relatives also deserve some time to rest.*

(APNAC_4)

The awareness raised among pre-service teachers, to which we have already referred, gives way to critical reflection upon essential issues, such as health or social wellbeing. These ideas are related to the effects of SL reported by Iverson and James [28], who assert that participant students increase their self-awareness in relation to others as well as their communities. Similarly, Seban [62] indicates that SL may generate changes in the students’ perceptions, understandings and beliefs about the social problems suffered by the service receivers; therefore, supporting our findings related to the development of judgement on political issues.

## 4. Conclusions

This paper has analyzed from a qualitative approach the implications of a SL program applied in the field of PE on pre-service teachers’ political perspectives. It is convenient to note, first, that the program was not focused on political issues nor applied in a political science course. Second, the program encompassed different social organizations, thus each group of students could develop their projects in divergent settings. Nevertheless, all of them attended children with Special Education Needs or/and at-risk. The direct service provided [39] placed students in a real social context; therefore, they were propelled to interact with a range of social actors (i.e., children, relatives, volunteers). Thus, pre-service teachers had to reflect upon their experience and, consequently, they developed their critical thinking.

Pre-service teachers realized that the personal, economic and material resources are insufficient for both the social organizations and the families of the children. Therefore, university students better understood the necessities and problems that service receivers must face day after day. This newly acquired knowledge generated discourses and attitudes focused on improving the social problems they had identified. Likewise, there was a greater sense of responsibility and civic engagement on the students, and their views on diversity and inclusion shifted to a positive lens. Finally, pre-service teachers highlighted the relevance of attending children with SEN, their families and the organizations that support them; and related these ideas to social justice conceptions. Bearing in mind the aforementioned findings; thus, we may conclude that the SL program emerged as an adequate tool to develop pre-service teachers’ political perspective.

In this sense, Harry and Salvador [25] consider that teaching is a political act by nature. Therefore, they assert that teacher educators should consider their public discourses and how they approach the political dimensions of teaching with pre-service teachers. Consequently, we may suggest that the SL program has promoted the students’ learning related to a political perspective and thus, has helped teacher educators to promote equity, democracy and social justice through the PE field PE [2].

This research does not come without limitations such as the sample or the specific context where the SL program was carried out. However, the findings let us suggest several interesting future research ideas. In this sense, we could triangulate our results with additional data gathered through interviews or other techniques in order to deepen into our findings. It may be interesting to analyze these data in the long-term too, to examine whether the intentions mentioned by the pre-service teachers become real actions. Additional possibilities could consist of developing different SL programs so that pre-service teachers could work with different types of children along the teacher education stage. To do so, teacher education plans should be organized and planned out adequately.

## Figures and Tables

**Table 1:** Overview of the SL program.

Service-Learning Program				
Subject	Specific Contents to be Worked in the Sessions	Academic Objectives	Service Objectives	Service Carried Out
Fundamentals of Body Expression; Motor Games in Early Childhood Education”. Annual subject in the 2nd year of the degree (6 credits ETCS)	Body schema, motor skills, coordination, muscle tone and body posture, expressing emotions, recognition of emotions, dance, motor games	To develop the ability to work on children’s motor skills and body expression through play and games. To promote the inclusion of students’ educational needs.	To improve the motor and socialization areas of children with educational needs. To improve the educational inclusion of students with educational needs.	Developing PE sessions related to corporal expression and motricity.

Source: Own elaboration.

## Data Availability

Not applicable.
